# Plasma cell-free DNA methylation: a liquid biomarker of hepatic fibrosis

**DOI:** 10.1136/gutjnl-2017-315668

**Published:** 2018-01-20

**Authors:** Buket Yiğit, Marie Boyle, Oğuz Özler, Nihan Erden, Faik Tutucu, Timothy Hardy, Christina Bergmann, Joerg H W Distler, Gupse Adalı, Murat Dayangaç, Derek A Mann, Mujdat Zeybel, Jelena Mann

**Affiliations:** 1 Department of Gastroenterology and Hepatology, School of Medicine, Koç University, Istanbul, Turkey; 2 Faculty of Medical Sciences, Institute of Cellular Medicine, Newcastle University, Newcastle upon Tyne, UK; 3 Department of Internal Medicine, University of Erlangen-Nuremberg, Erlangen, Germany; 4 Liver Transplantation Unit, Istanbul Bilim University, Florence Nightingale Hospital, Istanbul, Turkey

**Keywords:** liver, hepatitis B, hepatocellular carcinoma, hepatic fibrosis

We recently reported dynamic epigenetic markers of fibrosis detectable in patients’ plasma that may have utility in non-invasive diagnosis and staging of fibrosis in patients with chronic liver disease.^1^ Specifically, we uncovered DNA methylation markers at the human PPARγ promoter detectable in circulating cell-free DNA (ccfDNA) that display differential methylation densities. Remarkably, PPARγ hypermethylation correlated with progression to cirrhosis in alcoholic liver disease (ALD) and with specific stages of liver fibrosis in non-alcoholic fatty liver disease (NAFLD). Furthermore, ccfDNA signatures were traced back to the molecular pathology in fibrotic liver tissue, providing a biomarker of the underlying pathological process and defining hepatocytes as the source of hypermethylated DNA found in plasma.^1^

The original study posed several important outstanding questions: (1) Can ccfDNA methylation be used as a biomarker of fibrosis in liver diseases of other aetiologies? (2) Does the presence of hepatocellular carcinoma (HCC) alter the biomarker in plasma? (3) Does presence of fibrosis in other organs generate similar biomarker profiles?

**Figure 1:**
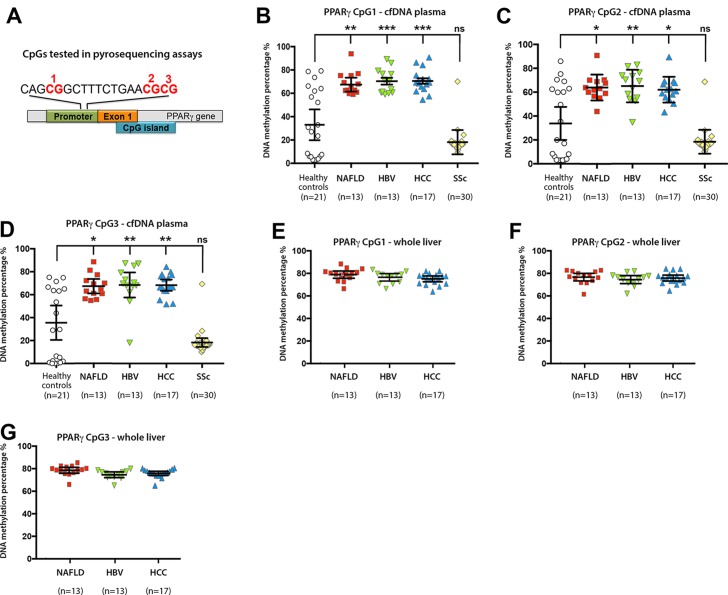
(A) Schematic representation of human PPARγ gene promoter showing the positions of the differentially methylated CpGs 1, 2 and 3. (B–D) Plasma cell-free DNA methylation as determined by pyrosequencing at (B) CpG1, (C) CpG2 and (D) CpG3 within the human PPARγ gene promoter from control donors or patients with NAFLD, HBV, HCC or SSc. n, shows the number of individual patients within each cohort. DNA methylation was quantitatively measured and expressed as a percentage. Error bars represent mean values±95% CI; *P<0.05, **P<0.01, ***P<0.001. (E–G) Whole liver DNA methylation at (E) CpG1, (F) CpG2 and (G) CpG3 within the human PPARγ gene promoter in patients with NAFLD, HBV and HCC. All methods are listed in the online supplementary file. cfDNA, cell-free DNA; HCC, hepatocellular carcinoma; NAFLD, non-alcoholic fatty liver disease; SSc, systemic sclerosis.

In the present letter, we answer these questions and demonstrate the broader utility of DNA methylation at three CpG dinucleotides within PPARγ promoter in several new patient cohorts (figure 1A and table 1). Employing pyrosequencing we detect hypermethylation at all three CpGs in ccfDNA from a cohort of patients suffering from cirrhosis caused by chronic HBV infection (figure 1B–D). The level of hypermethylation resembled that found in patients with cirrhotic NAFLD and ALD in our original study. However, since the HBV cohort was of another ethnicity to our original UK-based patients with NAFLD and ALD, we also measured methylation density in a Turkish NAFLD cohort, which was mirroring those detected in the HBV cohort. Our new data also demonstrate that presence of HCC with chronic liver disease does not alter the specificity of the DNA methylation markers for detection of liver fibrosis (figure 1B–D). As we had access to explant liver tissue from patients with NAFLD, HBV and HCC, we determined methylation densities in the liver (figure 1E–G). A high similarity was observed between the degree of DNA methylation at PPARγ gene promoter in ccfDNA and in the patient-matched liver tissues. We found a significant spread of values for DNA methylation in the healthy control ccfDNA, this being in contrast with our original UK-based study in which low-level methylation density was consistent across individuals within the control group. We are unable to explain this wider spread of methylation densities in the Turkish cohort, but cannot rule out an undetected liver disease in the apparently ‘healthy’ controls that display elevated ccfDNA methylation.

10.1136/gutjnl-2017-315668.supp1 Supplementary file 1

**Table 1:** Characteristics of patient cohorts used in the study

	Age (years)	Gender (male/female)	BMI (kg/m^2^)	Diabetes (%)	ALT (IU/L)	AST (IU/L)
NAFLD cohort	56 ± 7	10/3	29.8±3.2	69	33±23	54±36
Hepatitis B cohort	51±7	10/3	26.5±2.4	38	47±50	80±64
HCC cohort	57±7	16/1	27.5±4.2	29	55±36	65±53

Systemic sclerosis cohort (n=30)	Age (years)	Gender (male/female)	BMI (kg/m^2^)	Diffuse cutaneous limited SSc	Disease duration (years)	Heart involvement	Lung involvement	DLCO (%)	Antinuclear antibody-positive	Anticentromere antibody-positive	Antitopoisomerase I antibody-positive
	55±14	10/20	26±3.8	12 (40%)	7.5±4	2 (7%)	11 (37%)	71.7±17	30 (100%)	11 (37%)	12 (40%)

Notes: Viral hepatitis in HCC cohort: HBV-positive, n=8; HCV-negative, n=2; HBV-positive and HCV-positive, n=3.

Data expressed as mean±SD or median (range).

BMI, body mass index; HCC, hepatocellular carcinoma; NAFLD, non-alcoholic fatty liver disease; SSc, systemic sclerosis.

We next determined if hypermethylation is specific to fibrosis of liver origin. To this end, we quantified ccfDNA methylation in a cohort of patients with limited and diffuse systemic sclerosis (SSc) who have various combinations of skin, lung and kidney fibrosis, but no hepatic fibrosis.^2^ All three CpG sites in SSc were relatively hypomethylated (figure 1B–D), with similar methylation densities between individual patients with SSc. All methods relating to the study are listed in ‘online supplementary materials and methods 1’.

This important validation study supports our original hypothesis that hypermethylation at the PPARγ gene promoter is a marker for fibrotic progression of chronic liver disease and holds true for viral, alcoholic and metabolic disease aetiologies. As fibrosis in other organs does not generate a similar epigenetic signature, it is likely that the PPARγ hypermethylation specifically reflects a liver pathology. The ability to detect and quantify hypermethylation at the promoter of the PPARγ in ccfDNA as a new liquid biomarker that specifically reports the fibrotic progression of liver diseases of multiple aetiologies offers the potential for a cost-effective blood-based liquid biomarker of liver fibrosis.
