# Supplementary material for: Plasma cell-free DNA methylation: a liquid biomarker of hepatic fibrosis
Source: Gut. 2018 Jan 20;67(10):1907–8. doi: 10.1136/gutjnl-2017-315668 (PMC6145292; doi:10.1136/gutjnl-2017-315668)
Supplement: Supplementary file 1 [file gutjnl-2017-315668supp001.pdf]

Supplementary materials and methods to the letter "Plasma cell free DNA methylation: a liquid biomarker of hepatic fibrosis"

Clinical Cohorts

Use of human tissue was approved by Koç University Ethics Committee for Clinical Research (04.02.2016 - 2016.024.IRB2.005). Informed consent was obtained from all participants. All liver samples were collected and used subject to patient's written consent prior to the day of liver transplantation. Blood samples and liver tissues were collected during transplantation. Clinical and laboratory data were collected at the time of surgery.

NAFLD cohort- The NAFLD diagnosis was based on imaging findings and histologic examination of the explanted liver. Patients with alternative diagnosis (chronic viral hepatitis, viral autoimmune liver disease, drug-induced liver injury, haemochromatosis, Wilson's disease, alpha-1-antitrypsin deficiency) were excluded. Patients who consumed more than 20 g of alcohol per day for males or more than 10 g per day for females were excluded.

HBV and HCC cohort- For the HBV cohort, patients were selected based on the medical records with a positivity for hepatitis B surface antigen; patients with alternate liver diagnoses or evidence of coexistent liver disease were excluded. Diagnosis of HCC was made if typical features were detected on computed tomography/magnetic resonance and by pathologic examination. Severity of fibrosis was assessed on explanted liver tissues.

Clinical details such as gender, age, weight, height were obtained from all patients at the time of transplantation. The body mass index (BMI) was calculated by the

formula: weight (kg)/height^2 (m^2). Patients were identified as having type 2 diabetes if they were receiving dietary, oral hypoglycaemic drug or insulin treatment, or had fasting blood glucose >7.0 mmol/L. For the control cohort, use of human tissue was approved by Koç University Ethics Committee for Clinical Research (18.9.2015-2015.215.IRB1.020). Subjects had no signs or symptoms of liver disease, and no history of chronic illnesses.

Scleroderma Cohort - Systemic sclerosis (SSc) patients were recruited from a study site managed by Professor Jörg Distler, Professor for translational matrix biology, University of Erlangen-Nuremberg. Blood samples from patients were collected subject to patients' written consent. Recruited patients fulfilled the American College of Rheumatology (ACR)/ European League against Rheumatology (EULAR) criteria for the diagnosis of systemic sclerosis. SSc was classified according to the conventional criteria defined by LeRoy et al [1]. "Diffuse SSc" was diagnosed if the skin thickening extends proximal to the elbows and knees or includes the trunk, while "Limited SSc" was diagnosed if the skin thickening was confined to the elbows and knees, or to the face. Thirty SSc patients were recruited in total; eighteen had limited cutaneous SSc and twelve had diffuse cutaneous SSc. Information collected at time of blood sample collection involved clinical details (gender, age, weight, height, disease duration, organ involvement) and laboratory data (including Scleroderma related antibodies). The body mass index (BMI) was calculated by the formula: weight (kg)/height2 (m2). Lung involvement was considered present if there was evidence of pulmonary fibrosis or pulmonary arterial hypertension. Heart involvement was defined by a past/current diagnosis of congestive heart failure, cardiac arrhythmia, pericarditis, a pericardial effusion, or cardiomegaly.

Cell free circulating and liver DNA extraction

Whole blood was collected into EDTA and the plasma was separated by centrifugation for 10 min at 3000rpm followed by transfer to new tubes and re-centrifugation. For the chronic liver disease and hepatocellular carcinoma cohort, liver tissues were selected 3 cm away from tumour margin. Genomic DNA was extracted from plasma and liver specimens using QIAamp DNA Blood Mini or Micro Kit (Qiagen, Germany, catalogue no: 51106 - 56304). Plasma and liver tissues were lysed at 56°C for 10 minutes and overnight respectively. The lysate was processed and transferred to spin columns as per manufacturer's instructions.

Bisulfite modification

EZ DNA Methylation Gold TM Kit (Zymo Research, Irvine, CA, USA) was used for bisulfite conversion of genomic DNA. Cell free circulating and liver tissue DNA were bisulfite modified by incubating at 98°C for 10 min and 64°C for 2 h and 30 min. Product was transferred into columns; desulphonated and washed according to manufacturer's protocol and eluted in elution buffer. A 5µl of bisulphite modified cell free DNA was amplified in a PCR mix containing 2µl of forward and reverse primer, 12.5µl of HotStarTaq Master Mix Kit (Qiagen, Germany, catalogue no: 203445) or Pyromark PCR kit (Qiagen, Germany, catalogue no: 978703) and 5.5µl of water. 2.5µl Q solution and 1.5µl $MgCl_2$ (25mM/ml) were added. Amplification of DNA was performed in a thermocycler according to the following PCR conditions: one cycle at 95°C for 6 min, followed by 50 cycles of 95°C for 30 s, annealing temperature of 55°C for 30 s and 72°C for 30 s, followed by one cycle at 72°C for 30 s.

Pyrosequencing analysis

Methylation of specific cytosines within CpG dinucleotides was quantified by pyrosequencing using a Pyromark Q96 ID (Qiagen) instrument. PCR and sequencing primers were obtained from a custom designed assay for PPARγ as previously described [2]. 10μl of biotin-labelled PCR product was used in each well and combined by streptavidin- coated sepharose beads, washed in 70% ethanol, denatured in denaturation buffer (Qiagen, PyroMark Denaturation Buffer, 979007) and washed in a wash buffer (Qiagen, PyroMark Wash Buffer, 979008). Sequencing primers were annealed to DNA product at 80°C. The samples were analyzed in duplicate, and the mean of the two measurements was used as the final value. Assay efficiency was validated by fully unmethylated as well as fully methylated DNA (Qiagen, EpiTect PCR Control DNA Set, 59695). CpG methylation data was analysed by Pyro Q-CpG software 1.0.6.

Statistical analysis

All statistical analyses and graphs were made using GraphPad Prism Software. Continuous normally distributed variables were represented as mean ± standard deviation (SD). To determine differences between groups for continuous non-normally distributed variables, means were compared using the Mann-Whitney U test.

References

[1] LeRoy EC, Black C, Fleischmajer R, Jablonska S, Krieg T, Medsger TA, Jr., et al. Scleroderma (systemic sclerosis): classification, subsets and pathogenesis. The Journal of rheumatology 1988;15:202-205.

[2] Zeybel M, Hardy T, Wong YK, Mathers JC, Fox CR, Gackowska A, et al. Multigenerational epigenetic adaptation of the hepatic wound-healing response. Nature medicine 2012;18:1369-1377.
